# Multidisciplinary Orthodontic Management of Non-syndromic Delayed Tooth Eruption and Impacted Maxillary Canine Complicated by Traumatic Mandibular Fracture in a Healthy Adolescent: A Case Report

**DOI:** 10.1055/s-0046-1824590

**Published:** 2026-07-06

**Authors:** Miranda Sejdiu Abazi, Saranda Sejdiu Sadiku, Alban Olluri, Arben Abazi

**Affiliations:** 1 Department of Orthodontics, Faculty of Dentistry, UBT College, Prishtina, Kosovo; 2 Department of Biomedical Sciences, Universum College, Ferizaj, Kosovo; 3 Head of Department of Maxillofacial Surgery, Clinical Centre of Kosovo, Pristina, Kosovo; 4 Department of Maxillofacial Surgery, Clinical Centre of Kosovo, Pristina, Kosovo

**Keywords:** delayed eruption, canine impaction, fracture of mandible, orthodontic management

## Abstract

Delayed eruption of multiple teeth in systemically healthy, non-syndromic adolescents represents a diagnostic and therapeutic challenge. When combined with impacted maxillary canines and vertical discrepancies, management becomes more complex. A 14-year-old healthy male presented with delayed eruption (DE) of multiple permanent teeth. Clinical and radiographic examination revealed bilateral DE of mandibular canines and both premolars, a unilateral impacted maxillary canine, and a severe deep bite. The patient had no relevant medical history and showed normal physical development. Orthodontic treatment included space creation for mandibular teeth, closed surgical exposure of maxillary canines, and traction of the impacted maxillary canines, piggyback arch mechanics for canine alignment, and occlusal plane levelling using auxiliary intrusion mechanics supported by a mini-implant for skeletal anchorage. During treatment, the patient sustained a traumatic mandibular fracture, which required management and temporarily interrupted orthodontic therapy, prolonging overall treatment time. Following fracture stabilization, orthodontic treatment was resumed and completed, resulting in satisfactory functional and aesthetic outcomes. This case highlights the complexity of managing non-syndromic delayed tooth eruption combined with impacted canines and a deep bite, particularly when complicated by traumatic injury. Careful biomechanical planning and multidisciplinary coordination are essential for achieving stable outcomes.

## Introduction

Delayed eruption (DE) of permanent teeth may occur due to local or systemic factors. While syndromic and systemic causes are frequently reported, non-syndromic DE in otherwise healthy adolescents can present significant clinical challenges.

Impacted maxillary canines are among the most common eruption disturbances and often require combined orthodontic–surgical management. When associated with deep bite and multiple eruption delays, treatment planning becomes more complex and may require advanced biomechanical strategies, including skeletal anchorage.

This case report describes the multidisciplinary management of a healthy adolescent presenting with multiple DE and an impacted maxillary canine, complicated during treatment by a traumatic mandibular fracture.

Tooth eruption is a complex biological process involving the axial movement of a tooth from its intraosseous position into the oral cavity until functional occlusion is achieved. This process is regulated by genetic, local, and systemic factors and requires coordinated bone remodeling, periodontal ligament activity, and root development. 1 Recent studies have demonstrated that molecular signaling pathways and genetic factors play a crucial role in regulating the eruption process and are closely associated with eruption disturbances and clinical outcomes. 2 3 4

Eruption occurs in three principal phases—pre-eruptive, eruptive, and post-eruptive—and is closely related to root formation, with active eruption typically beginning when approximately two-thirds of the root has formed. 5 Contemporary evidence indicates that tooth eruption is governed by complex molecular signaling pathways and genetic regulatory mechanisms, which influence the timing and coordination of eruption and are implicated in the pathogenesis of eruption disturbances. 2 3 4 6

Minor variations in eruption timing are considered physiological; however, significant delay relative to the contralateral tooth or absence of eruption despite advanced root formation may indicate an eruptive disorder. 1 5 DE of permanent teeth may result from local, systemic, or genetic factors and should be differentiated from impaction and primary failure of eruption (PFE) through comprehensive clinical and radiographic assessment. 1 7 In the absence of mechanical obstruction, altered eruption timing may reflect developmental delay or disturbances in the eruption mechanism itself. 1 Early diagnosis and interceptive management are essential to minimize complications and optimize treatment outcomes. 7 Table 1 summarizes the important factors that contribute to the DE of mandibular teeth.

**Table 1:** Mandibular canines and premolars—delayed eruption

Tooth	Normal eruption age	Local causes	Systemic causes	Orthodontic management
Mandibular canine	9–10 years	• Lack of arch space • Retained primary canine • Deviated eruption path • Primary tooth ankylosis • Mechanical obstruction (supernumerary, odontoma)	• Delayed skeletal maturation • Vitamin D deficiency • Mild metabolic disorders	• Extraction of primary tooth • Space regaining (open coil, lingual arch) • 6–9 months monitoring • Surgical exposure + orthodontic traction if no eruption
First mandibular premolar	10–12 years	• Early loss of primary molar → space closure • Mesial migration of first molar • Retained primary molar • Ankylosis • Supernumerary/odontoma	• General delayed dental development • Hormonal factors	• Space regainer • Extraction of ankylosed primary tooth • Radiographic monitoring • Surgical exposure in rare cases
Second mandibular premolar	11–13 years	• Retained primary molar with incomplete resorption • Lack of space • Agenesis • Impaction due to molar tipping	• Delayed maturation • Familial factors	• Decision between retaining the primary tooth versus space opening • Fixed appliance treatment • Extraction in severe impaction cases

Maxillary canine impaction represents one of the most common eruption disturbances in orthodontic practice, with a reported prevalence of approximately 1 to 3%. 8 Space deficiency, ectopic eruption paths, and malocclusion patterns are frequently associated with canine impaction and may complicate orthodontic management. Table 2 summarizes the important factors that contribute to impaction of maxillary canines.

**Table 2:** Maxillary canine—causes of impaction

Category	Specific causes	Clinical/Radiographic indicators	Orthodontic considerations
Space deficiency	• Arch length deficiency • Severe crowding • Early loss of primary teeth	• Overlap with the lateral incisor root • Reduced arch perimeter	• Rapid maxillary expansion • Space creation with open coil springs • Anchorage reinforcement
Tooth position abnormalities	• Palatal displacement (most common) • Buccal displacement • Abnormal angulation (>15–20 degrees) • Ectopic tooth germ position	• Sector analysis (Ericson and Kurol) • Increased angulation on panoramic radiograph • CBCT confirmation	• Interceptive extraction (age 10–13) • Monitor 6–12 months • Surgical exposure + orthodontic traction
Associated dental anomalies	• Lateral incisor agenesis • Peg-shaped lateral incisor • Supernumerary teeth • Odontoma/cyst	• Missing or microdont lateral • Radiographic obstruction	• Removal of obstruction • Closed exposure technique preferred • Light-controlled forces (60–90 g)
Genetic/Familial factors	• Family history • Bilateral impaction • Associated dental anomalies	• Bilateral ectopic canines	• Early screening (age 9–10) • Multidisciplinary planning
Other contributing factors	• Trauma • Delayed skeletal maturation • Endocrine disturbances	• Dental age delay versus chronological age	• Individualized treatment timing • Growth monitoring

Radiographic assessment plays a critical role in identifying displacement and estimating the risk of impaction. 8 Recent studies have emphasized the importance of early diagnosis and screening in predicting maxillary canine impaction, highlighting the role of clinical and radiographic indicators as well as preventive orthodontic approaches in improving treatment outcomes. 9 10 11 When impaction is confirmed, surgical exposure followed by controlled orthodontic traction is widely accepted as the standard therapeutic approach. Clinical evidence demonstrates favorable periodontal outcomes with both open and closed exposure.

Vertical discrepancies such as deep bite often coexist with eruption disturbances and require anterior intrusion for correction. Conventional intrusion mechanics, as described by Burstone, employ light continuous forces delivered through segmented or utility arches to achieve controlled incisor intrusion. 12 This biomechanical approach emphasizes precise force magnitude and moment-to-force control to minimize unwanted tipping and reduce the risk of root resorption. Nanda further highlighted the importance of anchorage control and careful activation in achieving predictable vertical correction with auxiliary intrusion arches. 13 However, conventional mechanics depend on posterior dental anchorage and may produce reciprocal effects such as molar extrusion in cases of compromised anchorage.

The introduction of temporary anchorage devices (TADs), particularly orthodontic miniscrews, has significantly expanded biomechanical possibilities in complex cases. Miniscrews provide reliable skeletal anchorage independent of patient compliance and dental support, allowing direct force application to the anterior segment with minimal reciprocal effects. 14 Recent studies have further confirmed the clinical effectiveness, success rates, and biomechanical advantages of miniscrews in orthodontic treatment. 11 15 16 17 Their clinical application in anterior intrusion and deep bite correction has been well documented. 18 Systematic reviews and meta-analyses confirm the effectiveness and stability of miniscrew-supported mechanics, particularly in cases requiring absolute anchorage and enhanced vertical control. 19

Mandibular fractures are relatively common in children and adolescents, often resulting from trauma or accidents. 20 CBCT enables detailed three-dimensional visualization of mandibular structures, allowing precise identification of fracture lines, assessment of displacement, and evaluation of surrounding anatomical structures, thereby enhancing diagnostic accuracy and treatment planning. 21 22 When such injuries occur during active orthodontic treatment, they may interrupt biomechanical progression and necessitate temporary suspension of orthodontic forces. Interdisciplinary coordination between orthodontists and maxillofacial surgeons is essential to ensure proper skeletal healing while maintaining long-term orthodontic objectives.

The present case report describes the multidisciplinary orthodontic management of a healthy adolescent presenting with non-syndromic DE of multiple teeth and an impacted maxillary canine associated with a deep bite, further complicated by a traumatic mandibular fracture during treatment. This case highlights the importance of comprehensive diagnosis, biomechanical precision, anchorage control, and interdisciplinary collaboration in achieving stable functional and aesthetic outcomes in complex orthodontic scenarios.

## Case Report

A 14-year-old patient presented for orthodontic evaluation with the chief complaint of failure of eruption of the lower permanent teeth, despite exfoliation of the corresponding primary teeth at approximately 11 years of age. The patient and parents reported no family history of delayed tooth eruption or dental developmental anomalies. Due to persistent concerns regarding the absence of eruption, the patient was referred by a general dentist for specialist assessment.

Intraoral examination revealed the absence of both mandibular permanent canines and premolars in the dental arch. The overlying mucosa appeared clinically normal, with no evidence of swelling, inflammation, fibrosis, or mucosal thickening. In the maxillary arch, the permanent right maxillary canine was clinically absent, while the corresponding primary canine remained retained and *in situ* , without abnormal mobility. The contralateral maxillary canine was fully erupted and in functional occlusion.

Clinical palpation of the right maxillary canine region did not reveal a prominent labial bulge, suggesting a possible displaced or impacted position of the permanent canine. A bilateral class I molar relationship was observed. Additionally, a pronounced deep overbite was present, characterized by excessive vertical overlap of the maxillary incisors over the mandibular incisors, with substantial coverage of the buccal surfaces of the lower incisors.

Panoramic radiographic evaluation excluded dental agenesis and did not reveal any evident mechanical obstruction to eruption. The mandibular canines and premolars were present and appropriately positioned within the alveolar bone; however, insufficient arch space was noted, potentially compromising their eruption pathway.

Based on the patient's chronological age, clinical findings, and radiographic assessment, the absence of eruption of the mandibular canines and premolars was considered delayed relative to the expected eruption timing.

## Clinical Summary

(a) Bilateral DE of mandibular canines and premolars.
(b) Unilateral impacted right maxillary canine.
(c) Deep overbite.
(d) Space discrepancy.
(e) Bilateral class I molar relationship.
(f) No evidence of systemic abnormalities.
(g) No family history of DE.

Radiographic examination confirmed the presence of unerupted permanent teeth in the absence of any associated pathological lesions. Additionally, ectopic positioning of the maxillary right canine was observed, attributable to its aberrant spatial orientation within the alveolar bone ( Fig. 1 ).

**Fig. 1:**
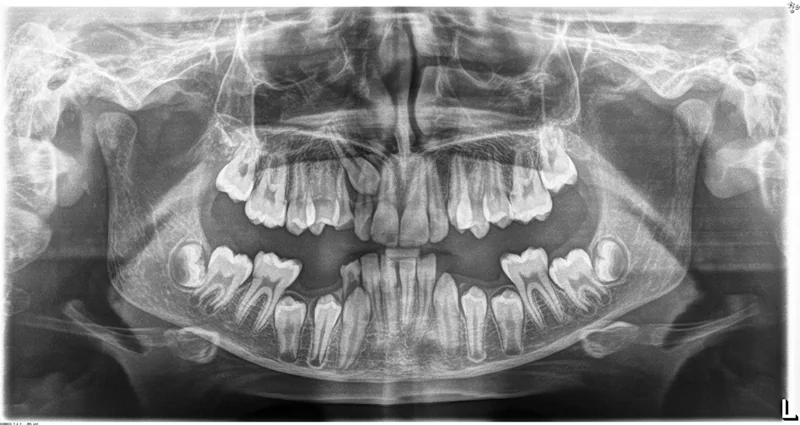
Pre-treatment panoramic radiograph demonstrating delayed eruption of mandibular canines and premolars, and impaction of the right maxillary canine.

In the present ortho-pantomogram, the canine crown is positioned within **sector 4** , indicating a mesial displacement that extends across the distal half of the maxillary central incisor. This sector location suggests an increased risk of impaction due to the medial migration of the canine crown.

The α angle (α) is increased, demonstrating a marked angulation of the long axis of the canine relative to the vertical mid-sagittal reference line. A greater α angle is associated with a less favorable eruption path and a higher likelihood of impaction.

Additionally, the vertical height (h) of the canine cusp tip relative to the occlusal plane is increased. The elevated vertical position further compromises the potential for spontaneous eruption.

Overall, the combination of a sector 4 position, increased α angle, and increased vertical height indicates an unfavorable prognosis for spontaneous eruption and suggests the need for interceptive or orthodontic intervention.

The radiographic measurements performed in Fig. 2 were based on the radiographic criteria described by Ericson and Karol, 8 including sector classification, α angle measurement, and vertical height assessment on panoramic radiographs. Radiographic evaluation of the bucco-palatal position of the impacted maxillary canine was conducted using cone-beam computed tomography. The dataset was analyzed in axial, coronal, and sagittal planes using multiplanar reconstruction. The bucco-palatal localization was determined based on the position of the canine crown relative to the alveolar ridge and dental arch. The findings indicated that the impacted canine was positioned palatally ( Fig. 3 ).

**Fig. 2:**
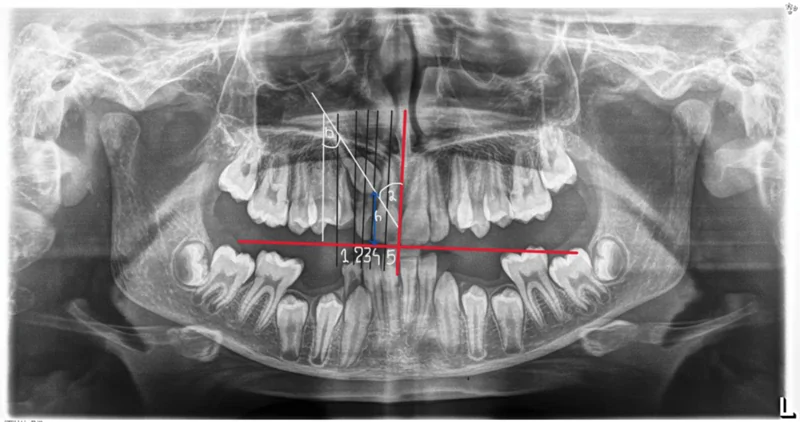
Radiographic criteria used to assess the position of the maxillary canine on a panoramic radiograph.

**Fig. 3:**
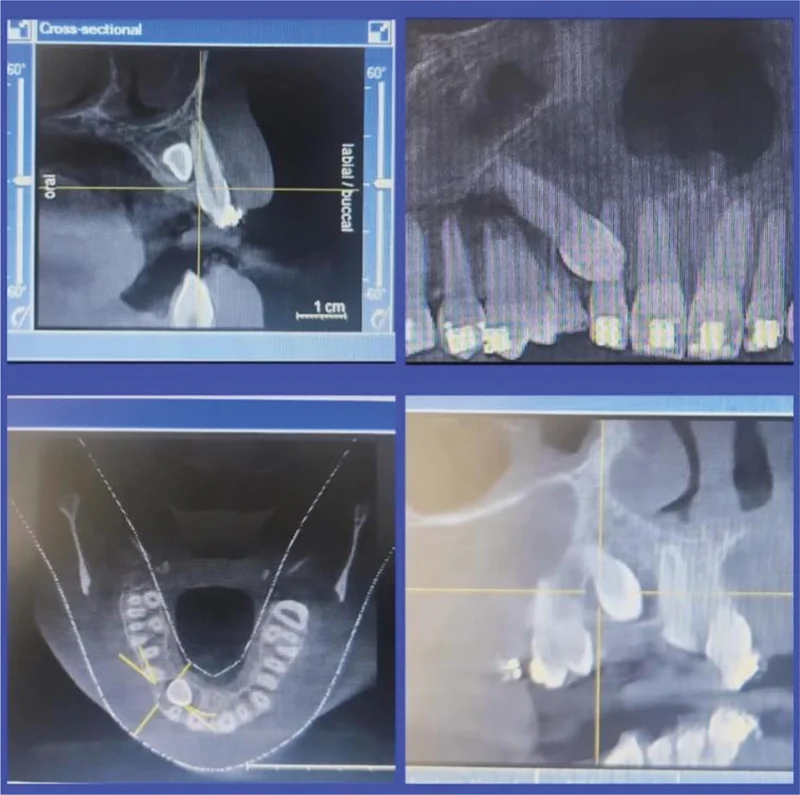
Radiographic evaluation of the bucco-palatal position of the impacted maxillary canine using cone-beam computed tomography (CBCT).

Space availability in the mandibular arch was assessed on panoramic radiographs by measuring the linear distance between the distal surface of the mandibular lateral incisor and the mesial surface of the first permanent molar. This segment represents the eruption pathway of the mandibular canines and premolars. The measured space was compared with the estimated mesio-distal width of the unerupted teeth to determine the presence of space deficiency ( Fig. 4 ). Although panoramic radiographs are subject to horizontal distortion and magnification, clinical examination also revealed reduced arch space, which was further confirmed by mixed dentition space analysis performed according to Moyers' analysis.

**Fig. 4:**
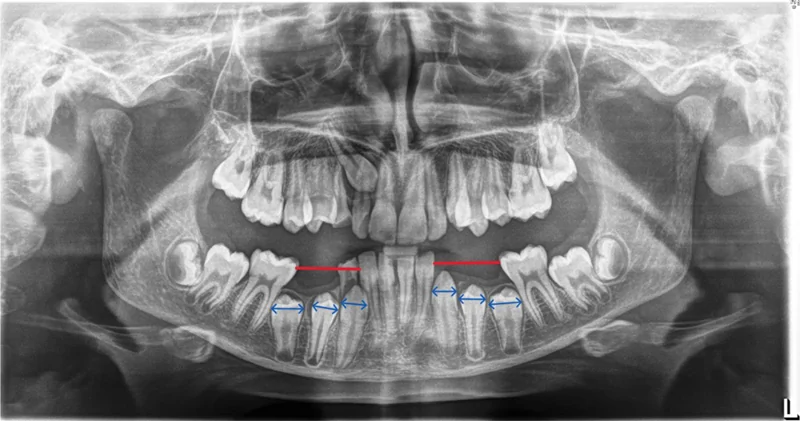
Radiographic evaluation of mandibular space discrepancy. The linear distance between the distal surface of the lateral incisor and the mesial surface of the first permanent molar was measured and compared with the estimated mesio-distal width of the unerupted teeth.

Available space – Required space = Space discrepancy

Right side:

20 mm − 28 mm = − 8 mm

Left side:

21 mm − 26 mm = − 5 mm

## Treatment Objectives

- Create sufficient space for the eruption of mandibular canines and premolars.
- Surgically expose and orthodontically align the impacted maxillary canine.
- Correct deep bite.
- Achieve functional occlusion and aesthetic alignment.

Initial photos at the beginning of the treatment are shown in Fig. 5 .

**Fig. 5:**
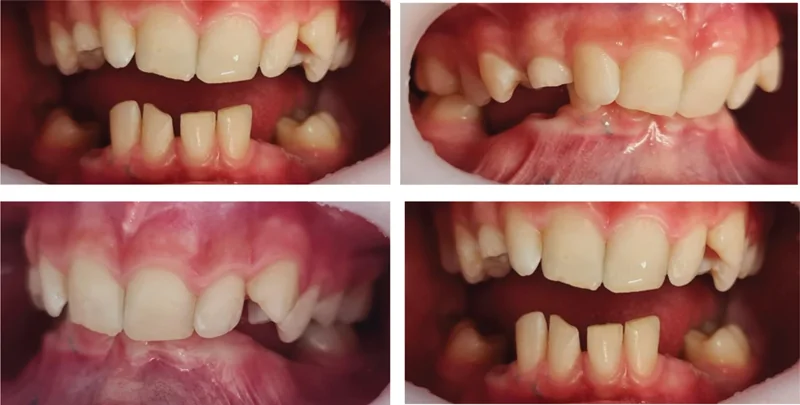
Intra-oral photos of the patient before initiation of orthodontic treatment.

## Treatment Progress

Orthodontic treatment was initiated with fixed appliances from Forestadent, Mini-Sprint II Brackets, “Roth System, with slot. 022,” with a 0.12 Ni-Ti arch wire. Space was created in the mandibular arch to facilitate the eruption of the mandibular canines and premolars with an open coil ( Fig. 6 ).

**Fig. 6:**
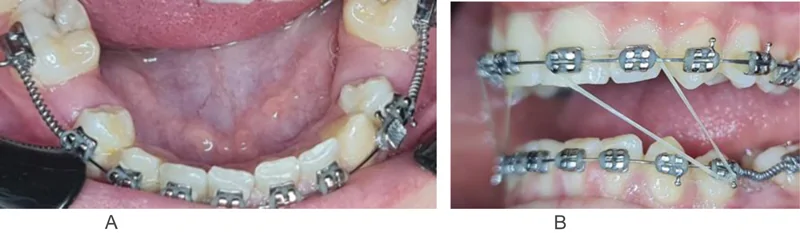
( **A** ) Space management with open coil. ( **B** ) Inter-arch elastics were applied to enhance occlusal relationships and facilitate space management.

The impacted maxillary canine was managed using a closed surgical exposure technique, followed by orthodontic traction. The maxillofacial surgeon raised the surgical flap, and at the same appointment, an orthodontic button with a gold chain (Forestadent) was bonded to the crown of the impacted canine. The flap was then repositioned and sutured, leaving only the terminal part of the chain accessible within the oral cavity to allow for orthodontic activation. Orthodontic traction was initiated after suture removal, ensuring adequate initial soft tissue healing.

Controlled eruption of the canine was achieved using piggyback arch mechanics. A rigid base arch-wire (e.g., stainless steel) was first placed in the main arch to provide anchorage and maintain arch form. Subsequently, a flexible auxiliary nickel–titanium (Ni-Ti) arch-wire was engaged over the main arch-wire (piggyback technique) and connected to the gold chain attached to the impacted canine. This configuration allowed the delivery of light, continuous, and controlled extrusive forces, minimizing unwanted side effects on adjacent teeth while guiding the canine into its correct position within the dental arch ( Figs. 7 and 8 ).

**Fig. 7:**
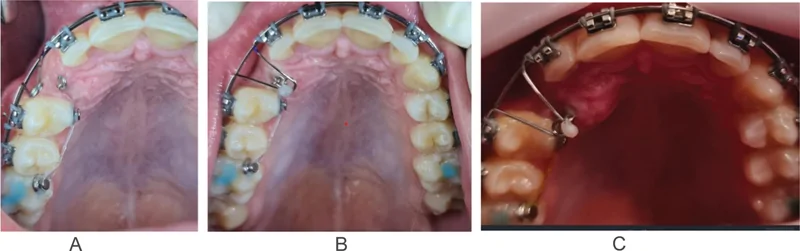
( **A, B** ) Piggyback arch mechanics used to achieve controlled alignment and guided eruption of the canine. ( **C** ) Treatment progress showing the cusp tip of the canine becoming clinically visible.

**Fig. 8:**
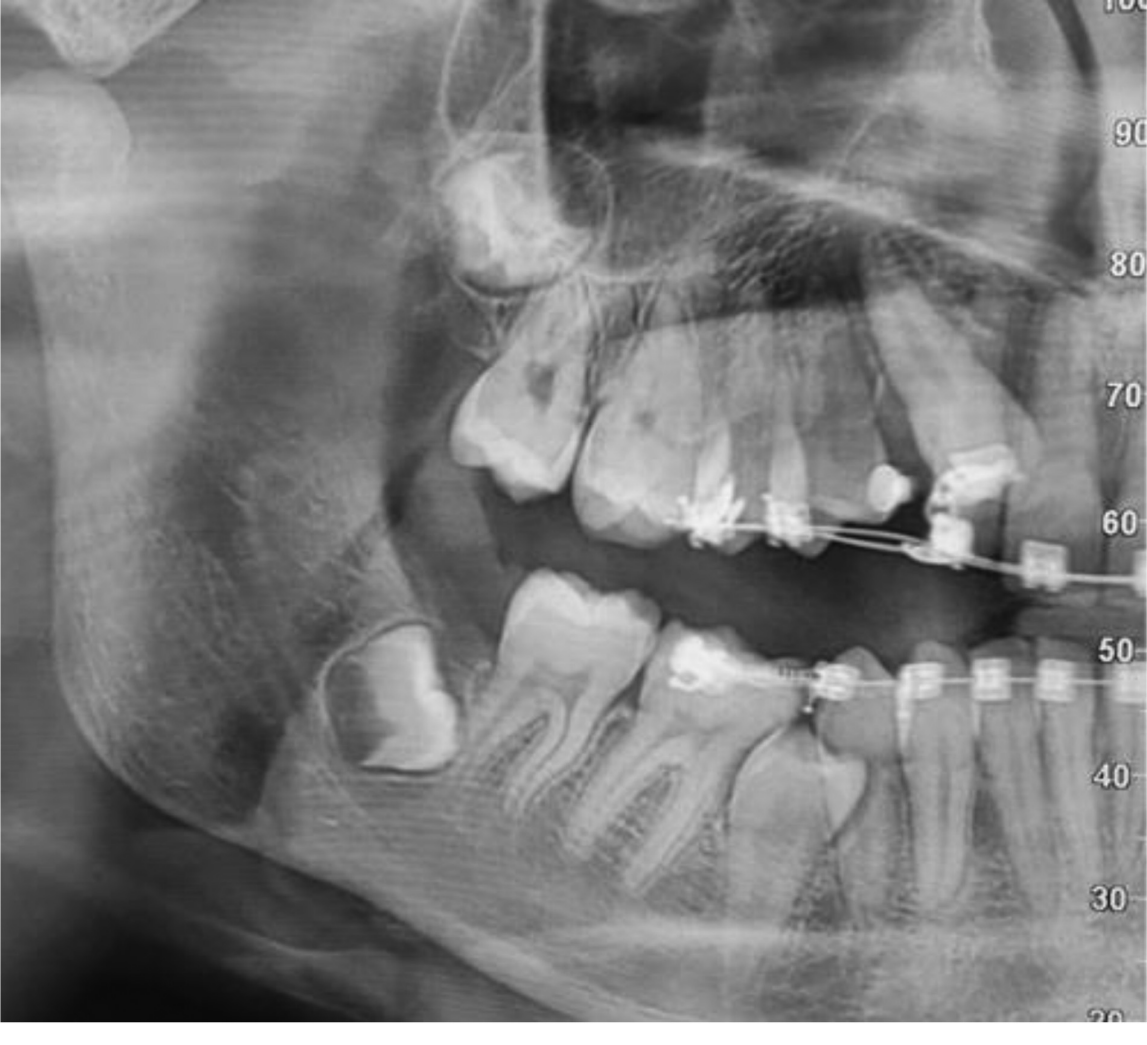
Follow-up panoramic radiograph illustrating orthodontic treatment progress in aligning the maxillary canine.

Deep bite correction and levelling of the maxillary occlusal plane were accomplished using auxiliary intrusion mechanics ( Figs. 9 10 11 ). Subsequently, a mini-implant (Forestadent diameter 1.7 mm, length 10 mm) was incorporated to provide skeletal anchorage, optimizing vertical control and overall biomechanical efficiency ( Fig. 12 ).

**Fig. 9:**
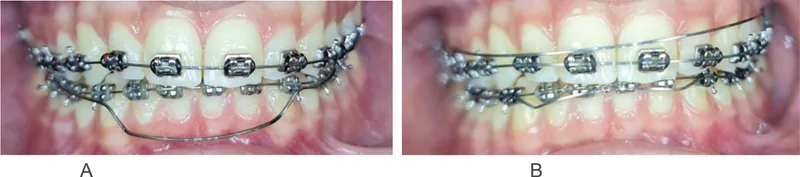
( **A, B** ) Intrusion arches.

**Fig. 10:**
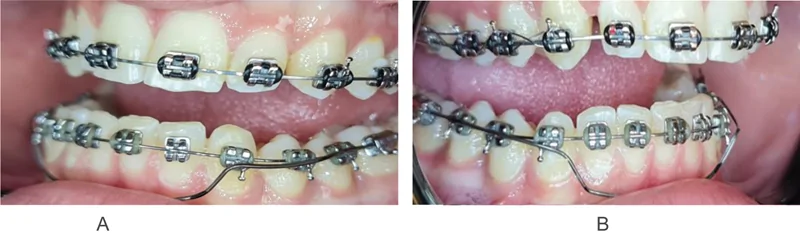
( **A, B** ) Mandibular intrusion arch before activation.

**Fig. 11:**
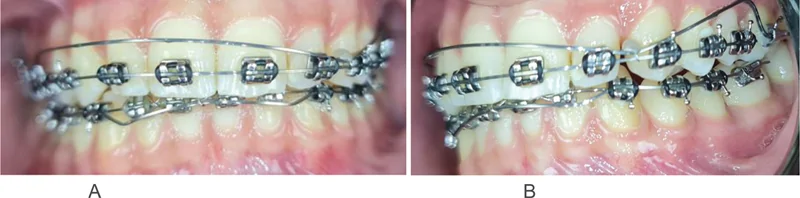
( **A, B** ) Maxillary intrusion arch before activation.

**Fig. 12:**
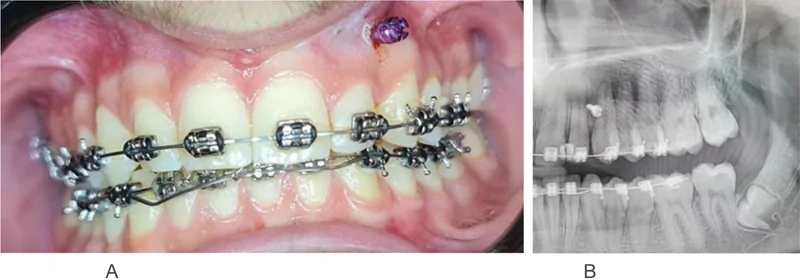
( **A** ) Mini-implant insertion, between the root of the left lateral incisor and canine. ( **B** ) Panoramic radiograph showing placement of a mini-implant to provide skeletal anchorage for anterior intrusion mechanics for occlusal plane canting.

During active treatment, the patient sustained a **traumatic mandibular fracture** while boxing, which required stabilization and temporary suspension of orthodontic forces ( Figs. 13 and 14 ).

**Fig. 13:**
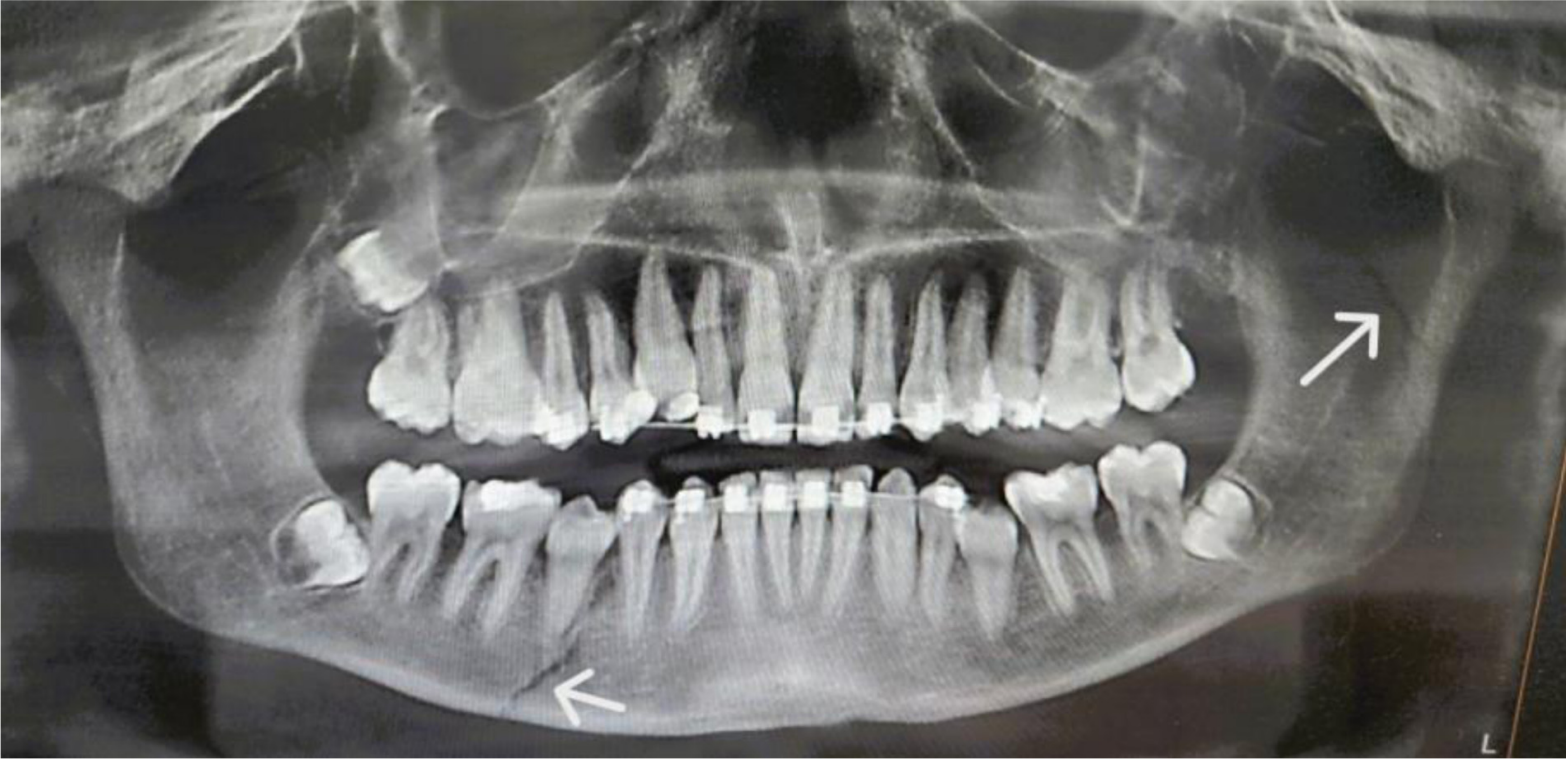
Radiographic image demonstrating fracture lines (indicated by arrows) secondary to trauma. A fracture of the right mandibular body is evident, with extension of the impact leading to an associated fracture of the condylar neck on the left side.

**Fig. 14:**
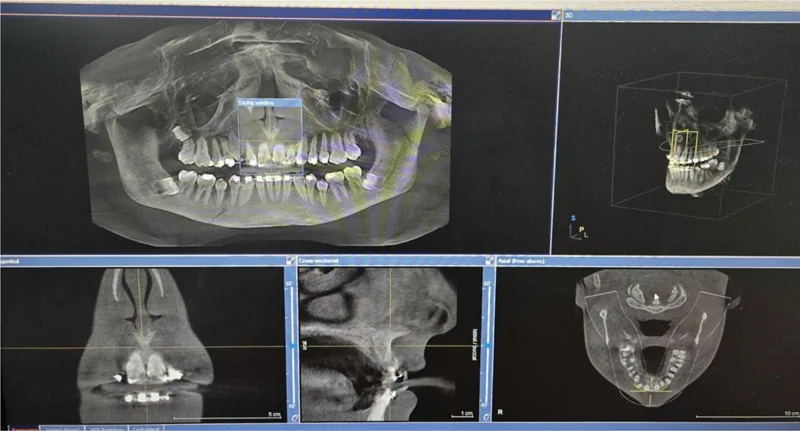
The fracture lines are also visible on CBCT.

Following appropriate healing and clearance, orthodontic therapy was resumed with modified mechanics to ensure safe continuation of treatment.

## Treatment Results

After treatment:

- Successful eruption and alignment of mandibular canines and premolars.
- Proper positioning of the maxillary canine.
- Correction of deep bite.
- Functional occlusion established.
- Acceptable facial aesthetics achieved ( Figs. 15 and 16 ).

**Fig. 15:**
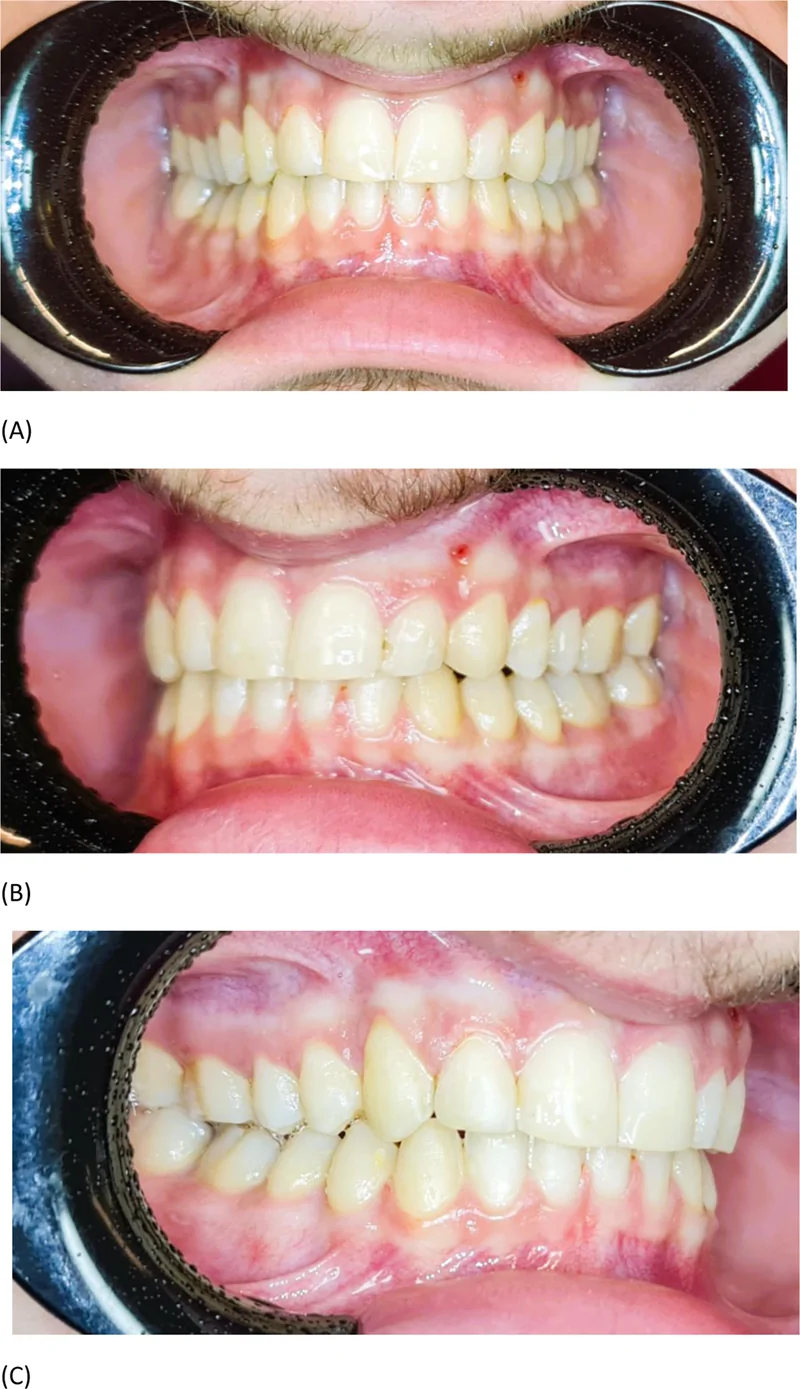
Final treatment: ( **A** ) Frontal view. ( **B, C** ) Lateral view.

**Fig. 16:**
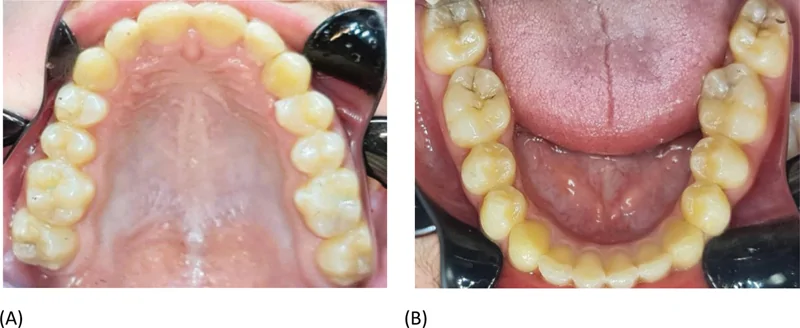
Final treatment: ( **A, B** ) Occlusal view.

The post-treatment cephalometric analysis demonstrates a skeletal class I relationship (ANB = 2 degrees) with well-balanced maxillary and mandibular positions (SNA = 82 degrees, SNB = 80 degrees). The vertical pattern is within normal limits, with improved mandibular plane inclination (SN–Go-Gn reduced to 25 degrees), indicating good vertical control ( Fig. 17 , Table 3 ).

**Fig. 17:**
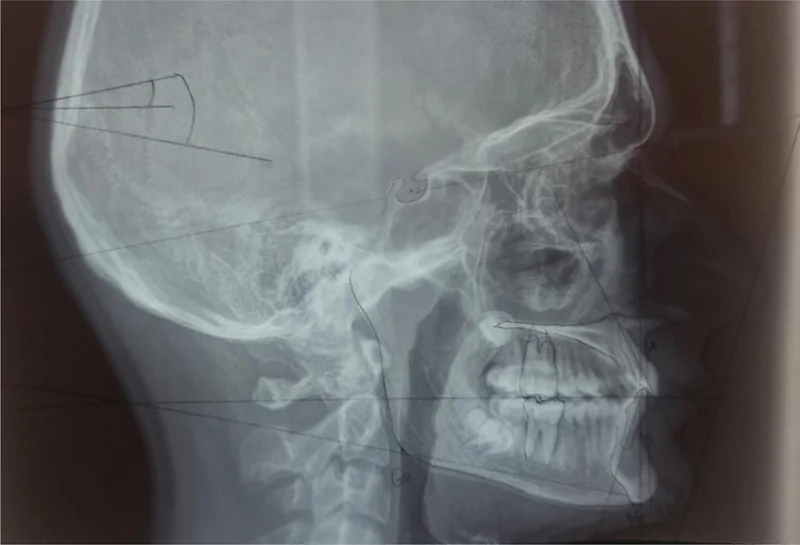
Post-treatment cephalometric analysis.

**Table 3:** Post-treatment cephalometric analysis

Measurement	Posttreatment value
SNA (°)	82
SNB (°)	80
ANB (°)	2
SN–Go-Gn (°)	25
U1–NA (°)	23
U1–NA (mm)	4
L1–NB (°)	21
L1–NB (mm)	3
Interincisal angle (°)	135

Dental measurements show proper incisor positioning (U1–NA = 23 degrees; L1–NB = 21 degrees) and a favorable inter-incisal angle, suggesting correction of the deep overbite without excessive proclination. Soft tissue evaluation reveals a slightly retrusive profile relative to the S-line. At the end of treatment, a mild residual rotation of the left mandibular first molar was observed. Further correction was discussed; however, the patient declined additional procedures due to treatment fatigue. Limited patient compliance throughout treatment, particularly in maintaining bracket integrity ( Table 3 ), contributed to a prolonged overall treatment duration of 42 months. This duration included a 6-month interruption secondary to a mandibular fracture, after which treatment was continued.

No significant root resorption or periodontal complications were observed ( Fig. 18 ).

**Fig. 18:**
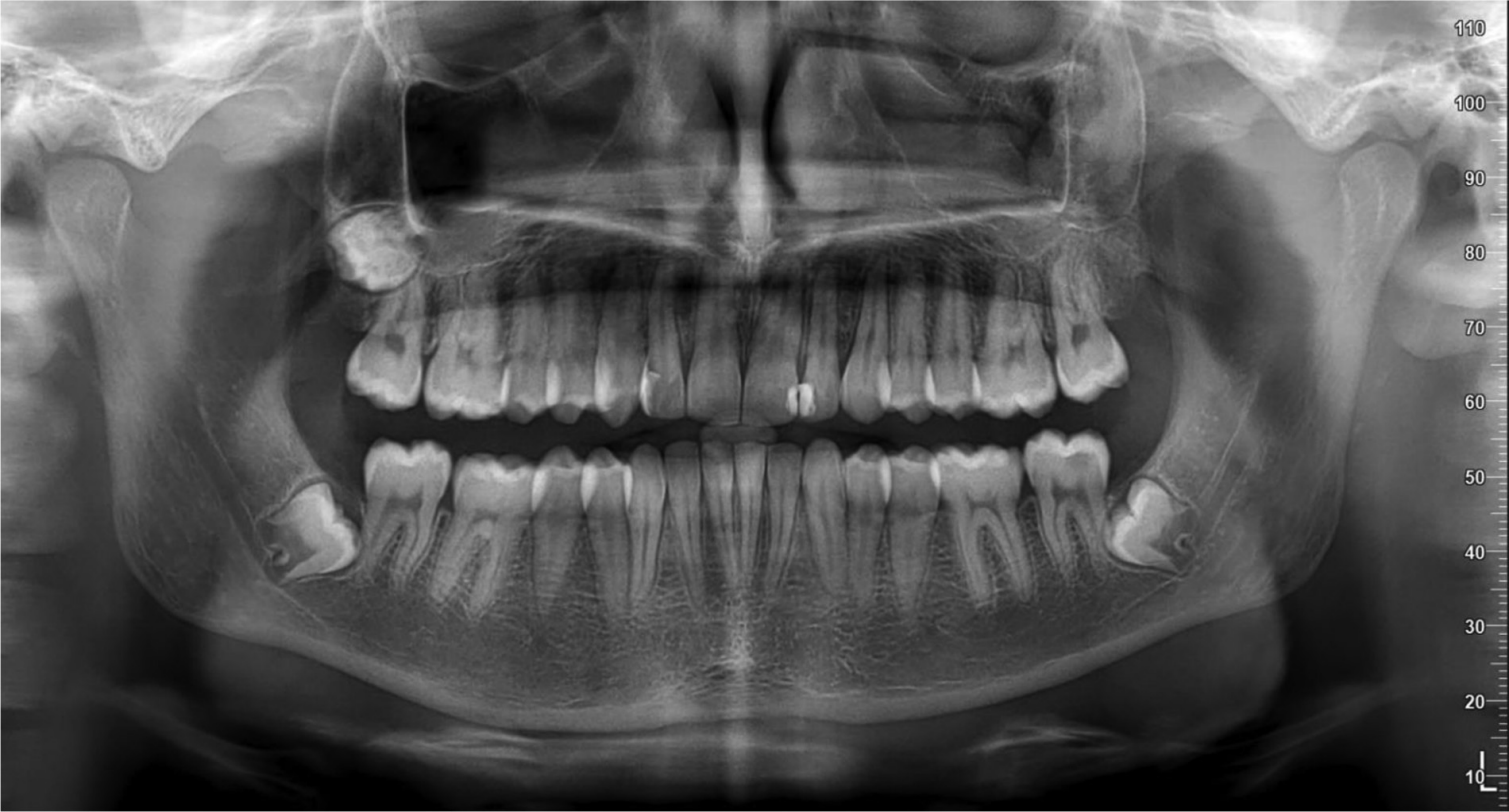
Post-treatment panoramic radiograph.

The patient was advised to undergo the extraction of third molars due to their unfavorable position.

## Discussion

DE of permanent teeth represents a significant diagnostic and therapeutic challenge, particularly when multiple teeth are involved in the absence of systemic or syndromic conditions. Non-syndromic DE affecting several permanent teeth is relatively uncommon and has been documented mainly in isolated case reports, emphasizing the need for comprehensive diagnostic evaluation in otherwise healthy individuals. 1 Careful differentiation between physiological delay, primary retention, and primary failure of eruption (PFE) is essential to prevent inappropriate treatment decisions. 1 3

Delayed tooth eruption may be associated with local factors such as retained primary teeth, ectopic eruption pathways, or space deficiency; however, in certain cases, no mechanical obstruction is identified. 2 3 4 7 23 When multiple teeth fail to erupt despite the absence of systemic disease, clinicians must consider disturbances in dental maturation or alterations in the eruption mechanism itself. 1 Early recognition is crucial, as a prolonged delay in eruption may adversely affect occlusal development and significantly influence orthodontic timing and overall treatment strategy. 23

The coexistence of an impacted maxillary canine further increases clinical complexity. Early radiographic assessment of maxillary canine position is essential to determine the likelihood of spontaneous eruption or the need for interceptive extraction. 8 Impacted canines are frequently associated with prolonged retention of primary teeth and abnormal positional relationships with adjacent incisors, potentially resulting in root resorption or arch asymmetry. 8 Their management commonly requires a combined surgical–orthodontic approach. Surgical exposure followed by controlled orthodontic traction has demonstrated favorable periodontal outcomes when appropriately executed, regardless of whether an open or closed exposure technique is used. 24 Recent studies further indicate that both open and closed surgical exposure techniques yield comparable clinical and patient-reported outcomes, with no significant differences in periodontal health when proper orthodontic mechanics are applied. 25 26 27

In cases where DE and impaction coexist, biomechanical planning and anchorage control become critical components of treatment.

Vertical discrepancies, including deep overbite, often accompany complex eruption disturbances and necessitate anterior tooth intrusion. Conventional intrusion mechanics, as described by Burstone, utilize light continuous forces delivered through segmented or utility arches to achieve controlled incisor intrusion. 12 This approach requires precise force calibration and adequate posterior anchorage to minimize undesirable tipping and reduce the risk of root resorption. 12 13 However, because intrusion arches rely on posterior dental anchorage, reciprocal effects such as molar extrusion or anchorage loss may occur, particularly in severe malocclusions or compromised anchorage conditions. 13

The introduction of TADs has significantly enhanced biomechanical control in orthodontic treatment. Mini-implants provide skeletal anchorage independent of dental support and patient compliance, allowing direct force application to the anterior segment. 14 This reduces unwanted reciprocal effects and improves vertical control, especially in cases requiring absolute anchorage. Evidence from systematic reviews and meta-analyses confirms the clinical effectiveness of mini-implant-supported mechanics, demonstrating improved anchorage stability and more predictable outcomes in complex tooth movements, including intrusion. 19

In the present case, the use of mini-implant anchorage provided effective vertical control and facilitated the intrusion mechanics necessary for deep bite correction and the canting of the occlusal plane. The occurrence of a traumatic mandibular fracture during treatment represented an unexpected clinical complication, necessitating temporary interruption of orthodontic forces and close interdisciplinary collaboration. This event highlights the importance of flexible treatment planning and coordinated multidisciplinary management in complex orthodontic cases. The integration of orthodontic and surgical approaches is essential in the management of mandibular fractures, with individualized treatment strategies and timely modification of orthodontic mechanics playing a key role in achieving optimal functional and clinical outcomes. 28 29 30 Clinical reports and recent case-based studies demonstrate that multidisciplinary management, combining orthodontic appliances with surgical or conservative approaches, can effectively stabilize mandibular fractures while allowing continued functional rehabilitation. The use of orthodontic techniques, such as bracket-assisted fixation, has been shown to support fracture management and improve patient comfort, while coordinated care between specialists ensures optimal healing and treatment outcomes. 31 32 33

Overall, management of non-syndromic DE involving multiple permanent teeth, combined with impacted maxillary canines and vertical discrepancies, requires an individualized and multidisciplinary approach. Accurate diagnosis, timely intervention, and careful selection of biomechanical strategy are fundamental to achieving stable functional and aesthetic outcomes while minimizing periodontal risks.

## Limitation

A limitation of this case was the absence of an initial lateral cephalometric radiograph, as such imaging was not available at our facility and required referral to another center. The patient did not proceed with the additional radiographic examination, which limited the comprehensive skeletal assessment.

## Conclusion

In this case, the multidisciplinary approach allowed successful management of DE and the impacted canine associated with previous trauma. Following detailed clinical and radiographic evaluation, space was created orthodontically, and closed surgical exposure of the impacted maxillary canine was performed. The tooth was subsequently guided into the arch using light and controlled orthodontic traction, with careful anchorage control throughout treatment.

Continuous monitoring was performed to assess periodontal and pulpal status, considering the history of trauma. The treatment resulted in proper alignment of the impacted canine, functional occlusion, and satisfactory aesthetic integration within the dental arch.

This case demonstrates that, with appropriate planning, controlled biomechanics, and close interdisciplinary collaboration, complex eruption disturbances can be successfully managed with stable outcomes.
